# Lower Circulating miR-122 Level in Patients with HNF1A Variant-Induced Diabetes Compared with Type 2 Diabetes

**DOI:** 10.1155/2018/7842064

**Published:** 2018-08-01

**Authors:** Xiuting Huang, Siqian Gong, Yumin Ma, Xiaoling Cai, Lingli Zhou, Yingying Luo, Meng Li, Wei Liu, Simin Zhang, Xiuying Zhang, Qian Ren, Yu Zhu, Xianghai Zhou, Rui Zhang, Ling Chen, Xueying Gao, Fang Zhang, Yanai Wang, Xueyao Han, Linong Ji

**Affiliations:** Department of Endocrinology and Metabolism, Peking University People's Hospital, Peking University Diabetes Center, Beijing, 100044, China

## Abstract

miR-122, the expression of which is regulated by several transcription factors, such as HNF1A, was recently reported to be associated with type 2 diabetes (T2DM) and hepatocellular carcinoma. HNF1A variants can cause diabetes and might be involved in the development of primary liver neoplasm. Differences in miR-122 expression among different types of diabetes have not been studied. This study aimed to investigate differences in serum miR-122 levels in Chinese patients with different forms of diabetes, including T2DM, type 1 diabetes (T1DM), HNF1A variant-induced diabetes (HNF1A-DM), glucokinase variant-induced diabetes (GCK-DM), and mitochondrial A3243G mutation-induced diabetes (MDM). In total, 12 HNF1A-DM patients, 24 gender-, age-, and body mass index-matched (1 : 2) T2DM patients and 24 healthy subjects were included in this study. In addition, 30 monogenic diabetes (11 GCK-DM and 19 MDM) and 17 T1DM patients were included. Fasted blood biochemistry and miR-122 were measured. The results showed that the HNF1A-DM patients had lower miR-122 levels [0.046 (0.023, 0.121)] than T2DM patients [0.165 (0.036, 0.939), *P* = 0.02] and healthy controls [0.249 (0.049, 1.234), *P* = 0.019]. The area under the curve of the receiver operating characteristic curve for miR-122 to discriminate HNF1A-DM and T2DM was 0.687 (95% CI: 0.52–0.86, *P* = 0.07). There was no difference in serum miR-122 among HNF1A-DM, GCK-DM, MDM, and T1DM patients. Lower serum miR-122 is a unique feature of HNF1A-DM patients and might partially explain the increased risk for liver neoplasm and abnormal lipid metabolism in HNF1A-DM patients.

## 1. Introduction

Diabetes mellitus (DM) is a chronic metabolic disorder characterized by progressive hyperglycemia and usually accompanied by lipid metabolism disorder. Maturity-onset diabetes of the young (MODY) is a rare monogenic form of diabetes mellitus that accounts for 2–5% of total diabetes cases and is often misdiagnosed as another type of diabetes [1–3]. The liver-enriched transcription factor (LETF) HNF1A is a transcriptional factor that is essential for liver differentiation and regulates a number of genes involved in lipid and glucose metabolism [4, 5]. It is well known that HNF1A variant-induced diabetes (HNF1A-DM) is the most common form of MODY.

MicroRNAs (miRs) are small noncoding RNA molecules that regulate gene expression at the posttranscriptional level [6]. miR-122 is one of the most abundant liver-specific miRs. It plays important roles in hepatocyte development, differentiation, and metabolism and is involved in several important aspects of liver pathophysiological processes, including fatty acid and cholesterol metabolism, hepatocarcinogenesis, and hepatitis C virus (HCV) replication [7–10]. In patients with chronic liver diseases, such as HCV infection and hepatocellular carcinoma (HCC), serum miR-122 levels are generally lower than those in healthy controls [11, 12]. Recently, miR-122 has been shown to have adverse metabolic effects in the general population. miR-122 levels were positively associated with the future development of insulin resistance, metabolic syndrome, and type 2 diabetes (T2DM) [13, 14]. Of note, miR-122 was shown to have a strong positive association with LETFs such as HNF1A and may be one of the downstream factors of this transcription factor [15–18]. Therefore, we hypothesized that serum miR-122 levels are reduced in HNF1A-DM patients and that low serum miR-122 is a potential biomarker for HNF1A-DM. In this study, we compared the levels of serum miR-122 among Chinese patients with different forms of diabetes, including HNF1A-DM, T2DM, type 1 diabetes (T1DM), glucokinase variant-induced diabetes (GCK-DM), and mitochondrial A3243G mutation-induced diabetes (MDM).

## 2. Materials and Methods

### 2.1. Study Participants

In total, 107 volunteers were recruited from in- and outpatients at Peking University People's Hospital, Beijing, China. Eighty-three DM cases included 11 GCK-DM, 12 HNF1A-DM, 19 MDM, 17 T1DM, and 24 T2DM patients. Twelve HNF1A-DM patients carried the heterozygous variants reported to cause diabetes (A311D, IVS8 + 1G > A, P379A, R131W, R200W, R229Q, R263C, T10M, or V567I). Eleven GCK-DM patients carried heterozygous variants reported to cause diabetes (V412E, S445R, R250C, Y234H, R186Term, R36W, R377H, G44S, or R43H). Eleven MDM patients carried mitochondrial tRNA^Leu(UUR)^A3243G mutation. If the patients had undetectable or low C peptide levels and persistently positive antibody associated with T1DM, they were diagnosed as having T1DM. In this study, T2DM was defined as fasting plasma glucose (FPG) ≥ 7.0 mmol/l and/or 75 g OGTT 2 h plasma glucose (2-hPG) ≥ 11.1 mmol/l (1999 World Health Organization criteria), a self-reported history of diabetes diagnosed by a physician or the use of antidiabetic agents. Twenty-four control subjects met the following criteria: (1) body mass index (BMI) < 24 kg/m^2^, (2) without hyperglycemia, hypertension, and other chronic diseases, (3) normal liver enzyme levels, alanine transaminase (ALT) < 50 U/L, aspartate aminotransferase (AST) < 50 U/L, and (4) no intake of any lipid-lowering drugs or alcohol. T2DM patients and healthy subjects were matched (1 : 2) according to gender, age, and BMI with HNF1A-DM patients in this study. All participants signed written informed consent. The study program was approved by the Ethics Committee of Peking University People's Hospital.

### 2.2. Biochemical Measurements and Clinical Data Collection

Blood and urine samples were collected from the study subjects after 8 h fasting. Genomic DNA was extracted from peripheral whole blood samples using the Blood DNA Mini Kit (SIMGEN, Hangzhou, China). Genotyping was conducted by polymerase chain reaction (PCR) amplification and Sanger sequencing. HbA1c, plasma glucose, ALT, AST, total cholesterol (TC), triglyceride (TG), high-density lipoprotein cholesterol (HDL-c), low-density lipoprotein cholesterol (LDL-c), and creatinine (CRE) levels and the urinary albumin/creatinine ratio (ACR) were determined as previously described [19]. Systolic and diastolic blood pressures were measured using a sphygmomanometer after resting for at least 5 min. Weight and height were measured to calculate the BMI by the formula: weight (kg)/height^2^ (m^2^). Waist and hip circumferences were also measured.

### 2.3. miR-122 Level Measurement by Quantitative Real-Time (RT-q) PCR

Serum miR-122 levels were measured as described previously [13]. Briefly, total RNA was extracted from plasma by using a miRNeasy Serum/Plasma Kit (Qiagen, Hilden, Germany) according to the manufacturer's specifications. RNA yield and integrity were assessed by using a NanoDrop 2000 spectrophotometer (Thermo Scientific, Waltham, MA, USA) and agarose gel electrophoresis and ethidium bromide staining, respectively. miR-122 expression was quantified by RT-qPCR. Reverse transcription was conducted in a 10 *μ*l reaction mixture and containing 50 ng RNA, 2 *μ*l PrimeScript Buffer (TaKaRa, Japan), 2 *μ*l RT primer (Applied Biosystems, Foster City, CA, USA), and 0.5 *μ*l PrimeScript RT Enzyme Mix I (TaKaRa) in a GeneAmp® PCR System 9700 (Applied Biosystems) at 37°C for 15 min, followed by 85°C heat inactivation for 5 s. The RT reaction mixture was stored at −20°C. qPCRs were run in a LightCycler® 480 II Real-time PCR Instrument (Roche, Switzerland), using 10 *μ*l PCR reaction mixtures containing 1 *μ*l cDNA, 3.5 *μ*l nuclease-free water, 5 *μ*l 2 × LightCycler 480 Probes Master (Roche), and 0.5 *μ*l TaqMan® microRNA Assay (catalog number 4427975; Applied Biosystems). Reactions were run in a 384-well optical plate (Roche) at 95°C for 10 min followed by 40 cycles of 95°C for 10 s and 60°C for 30 s. All reactions were conducted in triplicate. The miR-122 expression level was normalized to the level of Syn-cel-miR-39 and was calculated using the 2^−ΔΔCt^ method.

### 2.4. Statistical Analysis

Normally distributed continuous variables are presented as the means and standard deviations (SDs), and nonnormally distributed variables are presented as the medians and interquartile ranges (IQRs). Differences between groups were determined using Student's *t*-test for variables with a normal distribution. A *P* value < 0.05 was considered statistically significant. All statistical analyses were conducted using SPSS (Chicago, IL, USA) version 20.0.

## 3. Results

### 3.1. Clinical Features of and Serum miR-122 Levels in the Study Population

The clinical and biochemical characteristics of the patients and control subjects are presented in Table 1. Serum miR-122 levels were significantly lower in HNF1A-DM patients [0.046 (0.023, 0.121)] than in T2DM patients [0.165 (0.036, 0.939), *P* = 0.02] and healthy controls [0.249 (0.049, 1.234), *P* = 0.019]. To check whether HNF1A-DM patients could be discriminated from T2DM patients on the basis of serum miR-122, a receiver operating characteristic (ROC) curve was generated; the area under the ROC curve (AUC) for miR-122 was 0.687 (95% CI: 0.52–0.86, *P* = 0.07).

Serum miR-122 levels of GCK-DM, MDM, and T1DM patients were [0.086 (0.041, 0.357)], [0.094 (0.038, 0.440)], and [0.048 (0.026, 0.243)], respectively. There was no difference in serum miR-122 among HNF1A-DM, GCK-DM, MDM, and T1DM patients. When HNF1A-DM, GCK-DM, and MDM patients were pooled into one monogenic diabetes group, no differences in miR-122 levels were observed between this group and T1DM and T2DM patients and healthy controls. Lower LDL-c levels were observed in HNF1A-DM patients than in T2DM patients, while HDL-c levels were higher in female HNF1A-DM patients than in T2DM patients.

When HNF1A-DM, GCK-DM, MDM, T1DM, and T2DM patients were pooled into one diabetes group, there was no difference in serum miR-122 levels between diabetes patients [0.072 (0.035, 0.357)] and healthy controls [0.249 (0.049, 1.234), *P* = 0.112].

### 3.2. Clinical Features of HNF1A-DM and Non-HNF1A-DM Patients

When GCK-DM, MDM, T1DM, and T2DM patients were pooled into one non-HNF1A-DM group, it was observed that the LDL-c levels were significantly lower in HNF1A-DM patients (1.90 ± 0.77 mmol/l) than in non-HNF1A-DM patients [(2.44 ± 0.78 mmol/l), *P* = 0.045]. Further, HNF1A-DM patients were diagnosed earlier than non-HNF1A-DM patients. There were no significant differences in age, BMI, blood pressure, fasting insulin, C-peptide, HbA1c, HDL-c, triglyceride, creatinine, and ACR levels between the two groups. Linear regression analysis showed that the levels of miR-122 were negatively associated with diabetes type after adjustment for age, gender, BMI, HbA1c, and ALT (standardized beta coefficient = −0.234, *P* = 0.049).

## 4. Discussion

This study was the first to investigate differences in serum miR-122 levels in patients with different forms of diabetes. The results showed that serum miR-122 levels in HNFA-DM patients were lower than those in T2DM patients and healthy controls, while no differences were observed among T2DM, GCK-DM, MDM, and T1DM patients and healthy controls. Low serum miR-122 level was associated with HNF1A-DM, independent of age, gender, BMI, HbA1c, and ALT. Additionally, as expected, lower LDL-c levels were observed in HNF1A-DM patients than in patients with non-HNF1A-DM forms of diabetes, while HDL-c levels were higher than those in female T2DM patients. Thus, low serum miR-122 was a unique feature of HNF1A-DM patients that distinguished them from T2DM patients. The low serum miR-122 might partially explain the increased risk of liver neoplasm and abnormal lipid metabolism associated with HNF1A-DM.

A few of studies have demonstrated that miR-122 expression levels are positively associated with multiple LETFs, such as HNF1A, HNF3A, HNF3B, and HNF4A, which are involved in the differentiation of the liver and glucose and lipid metabolism [15, 17, 18, 20]. Wei et al. reported that HNF4A regulates gluconeogenesis and lipid metabolism through miR-122 expression [20]. However, Coulouarn et al. recently reported that HNF4A does not directly drive miR-122 expression because siRNA-mediated HNF4A silencing did not significantly alter miR-122 expression. In contrast, knockdown of HNF1A, HNF3A, or HNF3B resulted in reduced miR-122 expression, suggesting that miR-122 may be under the control of these transcription factors [15]. In fact, HNF4A acts upstream of HNF1A, mutation of which can cause a form of MODY (MODY1) [21]. Thus, this study confirmed that HNF1A directly regulates miR-122 expression.

Multiple studies have shown that miR-122 regulates cholesterol and fatty acid metabolism [13, 22–24]. Deletion of miR-122 in the liver resulted in significant decreases in total serum TG and cholesterol levels. Anti-miR-122 therapy reduces circulating cholesterol levels by downregulating cholesterol biosynthesis genes, including 3-hydroxy-3-methylglutaryl-coenzyme A synthase 1 *(HMGCS1)*, 3-hydroxy-3-methylglutaryl-coenzyme A reductase *(HMGCR)*, and phosphomevalonate kinase *(PMVK)* [25–27]. HNF1A-DM patients reportedly have lower LDL-c levels and higher HDL-c levels than T2DM patients [28, 29], and liver-specific knockout of HNF1A in mice resulted in low LDL-c levels, which might be attributed to decreased proprotein convertase subtilisin/kexin type 9 (PCSK9) and increased LDL receptor expression [30]. These findings indicate that HNF1A might regulate lipid metabolism in the liver via miR-122 expression. However, further studies are needed to confirm the relationships among HNF1A, miR-122, and lipid metabolism.

Several studies have revealed that primary liver tumors, including benign liver adenomatosis and HCC, are clustered in HNF1A-DM families [31–33]. HNF1A plays a key role in hepatocarcinogenesis. miR-122 expression is suppressed in human HCC, and downregulation of miR-122 is associated with metastasis and poor prognosis in HCC patients [34]. Therefore, it is possible that suppressed miR-122 expression might account for the association between HNF1A-DM and HCC.

Recent studies confirmed serum miR-122 is increased in T2DM patients and is associated with insulin resistance, obesity, metabolic syndrome, and T2DM [13, 14]. In this study, serum miR-122 levels were lower in HNF1A-DM than in T2DM patients and controls. Since miR-122 increases around the time of the onset of acute liver failure and liver damage or drug-induced hepatitis can promote elevated liver enzyme and miR-122 levels [35, 36], to identify a real association between HNF1A and miR-122, all subjects with elevated liver enzymes and patients receiving agents that affect the miR-122 level, such as statins, and patients consuming alcohol, were excluded in this study. Thus, it is not surprising that, despite the small sample size, this study could identify a difference in serum miR-122 between HNF1A-DM and T2DM patients. Unfortunately, the results of ROC analysis to distinguish HNF1A-DM and T2DM did not reach a statistical significance (AUC = 0.687, *P* = 0.07). A further study with a large sample size is needed.

This study had some limitations. Firstly, as mentioned above, the sample size was small. Because of the low prevalence of HNF1A-DM, cooperation among multiple centers would help in collecting more HNF1A-DM samples. However, the results of the current study do provide valuable clues for further research. Secondly, for a small sample size study, assay errors for a given sample should be avoided. Although a widely accepted method for measuring miR-122 was adopted in this study, more sensitive and accurate quantification methods, such as digital PCR, would help improve the reliability of the results.

In conclusion, this study showed that miR-122 expression is decreased in HNF1A-DM patients compared to T2DM patients and healthy subjects, which might partially explain the abnormal lipid metabolism and an increased risk for liver neoplasms in HNF1A-DM patients and help us understand the nature of HNF1A-DM. The results also provided clues to further explore the pathophysiology of HNF1A-DM and the clinical characteristics of HNF1A-DM patients.

## Figures and Tables

**Table 1:** Serum miR-122 and clinical features of the patients with different types of diabetes.

	NGT	GCK-DM	HNF1A-DM	MDM	T1DM	T2DM	Monogenic diabetes mellitus
Number	24	11	12	19	17	24	42
Gender, male, number (%)	8 (33.33)	7 (63.64)	4 (33.33)	9 (47.37)	9 (52.94)	8 (33.33)	20 (47.62)
Age, mean (SD) (years)	34.71 ± 7.62	49.27 ± 18.05	33.17 ± 16.50^§^	40.68 ± 12.14	37.35 ± 11.62	34.33 ± 14.18	40.79 ± 15.92
Diagnosis age, mean (SD) (years)	N/A	46.20 ± 18.78	24.17 ± 11.38^§,^^∗^^,†^	33.59 ± 8.96	33.29 ± 8.85	29.96 ± 11.57	32.12 ± 13.38
BMI, mean (SD) (kg·m^−2^)	21.66 ± 1.29	24.05 ± 4.10	22.09 ± 3.61	20.54 ± 3.11	22.21 ± 3.38	22.56 ± 3.10	21.90 ± 3.74
Waist circumference, mean (SD) (cm)							
Male	77.74 ± 3.39	87.70 ± 11.81	77.83 ± 20.10	75.67 ± 4.27	82.50 ± 7.68	82.00 ± 9.02	80.04 ± 11.35
Female	73.97 ± 4.20	78.00 ± 9.35	81.25 ± 8.45^‡^	73.00 ± 10.21	79.00 ± 12.10	79.56 ± 9.25	77.10 ± 9.72
History of hypertension, number (%)	0 (0)	5 (45.45)	3 (25.00)	5 (26.32)	2 (11.76)	3 (12.50)	13 (30.95)
SBP, mean (SD) (mmHg)	111.04 ± 11.04	129.27 ± 25.13	111.36 ± 14.56	118.39 ± 17.75	116.00 ± 15.42	117.92 ± 12.65	119.45 ± 20.00
DBP, mean (SD) (mmHg)	74.67 ± 7.42	75.18 ± 15.09	68.09 ± 6.83^‡^	73.00 ± 10.79	71.00 ± 9.97	70.79 ± 10.12	72.25 ± 11.34
Therapy							
OHA, number (%)	N/A	1 (9.09)	8 (66.67)	10 (52.63)	7 (41.18)	12 (50.00)	19 (45.24)
Insulin, number (%)	N/A	0 (0)	7 (58.33)	11 (57.89)	13 (76.47)	9 (37.50)	18 (42.86)
FPG, mean (SD) (mmol/l)	5.10 ± 0.36	6.66 ± 0.65	8.21 ± 3.51^‡^	8.61 ± 3.41	8.63 ± 3.55	8.36 ± 3.68	7.91 ± 2.96
HbA1c, mean (SD) (%)	5.15 ± 0.31	6.61 ± 0.27	8.93 ± 3.15^‡,§^	8.53 ± 2.52	9.45 ± 2.27	9.85 ± 3.02	8.06 ± 2.49
HbA1c, mean (SD) (mmol/mol)	32.79 ± 3.38	48.73 ± 2.99	74.07 ± 34.47^‡,§^	69.71 ± 27.50	79.75 ± 24.84	84.11 ± 33.03	64.63 ± 27.22
Triglyceride, median (IQR) (mmol/l)	0.65 (0.45, 0.83)	0.82 (0.59, 1.10)	1.30 (0.98, 1.70)^‡^	1.18 (0.97, 1.76)	1.01 (0.64, 1.42)	1.12 (0.86, 1.89)	1.12 (0.83, 1.64)
LDL-C, mean (SD) (mmol/l)	2.37 ± 0.70	2.27 ± 0.83	1.90 ± 0.77^||,^^∗^	2.76 ± 0.63	2.14 ± 0.76	2.56 ± 0.79	2.35 ± 0.80
HDL-C, mean (SD) (mmol/l)							
Male	1.18 ± 0.17	1.31 ± 0.33	1.05 ± 0.43	1.09 ± 0.38	1.12 ± 0.29	0.98 ± 0.08	1.17 ± 0.36
Female	1.48 ± 0.30	1.60 ± 0.45	1.77 ± 0.68^||^	1.35 ± 0.28	1.49 ± 0.79	1.16 ± 0.36	1.61 ± 0.54
Fasting C peptide, mean (SD), (*μ*U/ml)	N/A	N/A	1.51 ± 0.58^†^	1.29 ± 0.61	0.66 ± 0.53	1.75 ± 0.98	1.36 ± 0.59
ALT, mean (SD) (U/l)	17.75 ± 6.39	17.64 ± 7.38	23.85 ± 15.90	21.75 ± 12.30	15.76 ± 7.53	22.00 ± 11.46	21.15 ± 12.26
AST, mean (SD) (U/l)	21.04 ± 5.65	18.91 ± 3.18	18.74 ± 5.78	18.69 ± 6.61	16.24 ± 4.35	22.17 ± 7.72	18.77 ± 5.39
CRE, mean (SD) (*μ*mol/l)	55.25 ± 10.77	67.91 ± 14.40	64.58 ± 33.07	66.31 ± 22.02	58.88 ± 15.98	52.79 ± 12.45	66.27 ± 23.49
UA, mean (SD) (umol/l)	250.15 ± 64.86	318.45 ± 68.31	288.73 ± 57.23	307.46 ± 87.12	278.71 ± 90.19	318.83 ± 66.86	305.03 ± 71.84
ACR, median (IQR) (mg/g)	3.58 (1.11, 7.70)	3.84 (2.50, 15.61)	7.66 (3.56, 15.26)	10.56 (1.59, 55.12)	5.09 (3.51, 10.93)	5.38 (3.85, 11.68)	7.40 (2.50, 18.34)
miR-122, median (IQR)	0.249 (0.049, 1.234)	0.086 (0.041, 0.357)	0.046^‡,||^ (0.023, 0.121)	0.094 (0.038, 0.440)	0.048 (0.026, 0.243)	0.165 (0.036, 0.939)	0.083 (0.034, 0.172)

Normally distributed continuous variables are presented as the means and standard deviations (±SDs), and nonnormally distributed variables are presented as the medians (interquartile range (IQR)). Categorical variables are presented as the numbers and percentages. Differences between groups were compared by Student's *t*-test or Chi-square test as appropriate. Nonnormally distributed variables were subjected to a natural logarithm transformation to obtain a normal distribution prior to statistical analysis. A *P* value < 0.05 was considered statistically significant.^||^Comparison between the HNF1A-DM and the T2DM groups (*P* < 0.05). ^‡^Comparison between the HNF1A-DM and the NGT groups (*P* < 0.05). ^§^Comparison between the HNF1A-DM and the GCK-DM groups (*P* < 0.05). ^∗^Comparison between the HNF1A-DM and the MDM groups (*P* < 0.05). ^†^Comparison between the HNF1A-DM and the T1DM groups (*P* < 0.05). NGT: normal glucose tolerance; T1DM: type 1 diabetes mellitus; T2DM: type 2 diabetes mellitus; MDM: mitochondrial diabetes mellitus; N/A: not available; BMI: body mass index; OHA: oral hypoglycemic agents; SBP: systolic blood pressure; DBP: diastolic blood pressure; FPG: fasting plasma glucose; HbA1c: glycated hemoglobin; LDL-C: low-density lipoprotein cholesterol; HDL-C: high-density lipoprotein cholesterol; TG: triglyceride; ALT: alanine transaminase; AST: aspartate aminotransferase; UA: uric acid; CRE: serum creatinine; ACR: urinary albumin/creatinine ratio.

## Data Availability

Data sharing is not applicable for this article.
